# PRISEC: Comparison of Symmetric Key Algorithms for IoT Devices

**DOI:** 10.3390/s19194312

**Published:** 2019-10-05

**Authors:** Daniel A. F. Saraiva, Valderi Reis Quietinho Leithardt, Diandre de Paula, André Sales Mendes, Gabriel Villarrubia González, Paul Crocker

**Affiliations:** 1 Departamento de Informática, Universidade da Beira Interior, 6201-601 Covilhã, Portugal; 2 Instituto de Telecomunicações, Delegação da Covilhã, 6201-601 Covilhã, Portugal; 3 Laboratory of Embedded and Distributed Systems-LEDS, Universidade do Vale do Itajaí, Itajaí, SC 88302-901, Brazil; 4 Computer and Automation Department, University of Salamanca, Plaza de la Merced s/n, 37008 Salamanca, Spain

**Keywords:** cryptography, IoT, privacy, UbiPri

## Abstract

With the growing number of heterogeneous resource-constrained devices connected to the Internet, it becomes increasingly challenging to secure the privacy and protection of data. Strong but efficient cryptography solutions must be employed to deal with this problem, along with methods to standardize secure communications between these devices. The PRISEC module of the UbiPri middleware has this goal. In this work, we present the performance of the AES (Advanced Encryption Standard), RC6 (Rivest Cipher 6), Twofish, SPECK128, LEA, and ChaCha20-Poly1305 algorithms in Internet of Things (IoT) devices, measuring their execution times, throughput, and power consumption, with the main goal of determining which symmetric key ciphers are best to be applied in PRISEC. We verify that ChaCha20-Poly1305 is a very good option for resource constrained devices, along with the lightweight block ciphers SPECK128 and LEA.

## 1. Introduction

With the rapid growth of the IoT (Internet of Things), more devices are connected to the Internet, resulting in bigger data exchanges. In turn, this generates more security and privacy risks for the users of these devices, which is currently one of the biggest challenges of the IoT [1,2,3]. Another problem comes from the fact that IoT devices are often limited in terms of computing power, energy, and memory capacity. The standard Internet protocols and cryptography algorithms require many of these resources, which can potentially make them unsuitable for IoT devices [4]. To deal with these problems, lightweight block ciphers can be used to protect data [5]. There is also a lack of standards for heterogeneous technologies and limited resource environments, which is the case of IoT devices. This opens further privacy risks and makes the IoT especially vulnerable to DDoS (distributed denial of service) attacks [6].

A popular protocol in the IoT is CoAP (constrained application protocol). It is intended to be used in limited resource environments, which makes it a good choice for IoT devices. It is a customized and compressed version of HTTP (hypertext transfer protocol). However, CoAP is susceptible to many types of attacks as studied in [7], including but not limited to parsing attacks (where a remote node can be crashed by executing arbitrary code), amplification attacks (an attacker can use end devices to convert small packets into larger packets), and spoofing attacks. This shows how IoT protocols still have many vulnerabilities, and it is becoming increasingly important to protect them against attacks.

In [8], the authors introduced a Cloud-based IoT architecture along with a series of security and privacy requirements to ensure the safety of data. These requirements included identity privacy (the user’s real identity has to be protected from the public), location privacy (the user’s location has to be protected as to not disclose their living habits), node compromise attack (to prevent an attacker from extracting private data from the devices), layer-removing/adding attack (to mitigate packet forwarding attacks), forward and backward security (meaning that new users can only decipher encrypted messages after joining the cluster and that revoked users cannot decipher encrypted messages after leaving), and semitrusted and/or malicious cloud security (meaning that input, output, and function privacy must be achieved). In [9], a privacy-preserving outsourced calculation toolkit was proposed for Cloud-based IoT. The main goal was to allow its users to outsource their data in cloud storage in a secure manner. A fully homomorphic encryption scheme was used, achieving efficient integer computations on encrypted data. These works took important steps in ensuring the safety of data in Cloud-based IoT.

The IoT has also been making its way to e-health systems, allowing a more efficient monitoring of patients with severe illnesses. The work developed in [10] analyzed the challenges of preserving the privacy in these systems. To handle these issues, a fusion of IoT and big data was designed to construct a system to secure communications and confidential medical data. An authenticated key distribution procedure was modeled for use in the medical network along with an algorithm which verifies the source of encrypted messages. The tests showed that this system is more efficient than other related works. The same authors developed in [11] a smart IoT-based healthcare big data storage with self-adaptive access control. Unlike other related systems, it combines attribute-based encryption to achieve fine-grained access control over encrypted data, cross-domain to allow several medical institutes to be in the network and share medical files, break-glass access to provide emergency access to encrypted medical files when the owner’s authorization is not present, and a password-based break-glass key, which is preset by the patient and a contact holds it for emergency situations when break-glass access has to be activated.

Another good way to solve the security and privacy problems in the IoT is through the use of middleware. Middleware can be defined as an interface between the hardware and the application with the main goal of managing the problem of heterogeneity. This way, the applications can run on many different devices and apply similar protocols and standards to all of them, enhancing security, performance, and reliability. Many middleware solutions have been developed over the years with the goal of standardizing the IoT [12]. However, a big number of these solutions still have problems related to security and privacy. A survey made in [13] analyzed 10 middleware solutions and found that four did not address security and privacy. Similarly, in [14], 22 middleware solutions were studied, and it was verified that 12 did not have a security model defined. Furthermore, 14 of the solutions did not have a tangible security architecture.

In [15], the middleware UbiPri (ubiquitous privacy) was developed with the main goal of managing and controlling the privacy of its users in ubiquitous environments automatically. Users are given an access level when they enter a new environment taking into account several factors, including but not limited to time of the day, if it is a working day or if the environment is public or private.

A practical example of UbiPri could be its users entering a theater to watch a play. Being a public environment where noise and interruptions are undesirable, the middleware would grant a low access level to the users and automatically silence their devices, block notifications or even limit their access to the Internet. Another example could be the apartment of a user. The apartment would be a private environment and the user its owner; therefore, they would have the highest access level (*Admin*). If the user receives guests in their apartment, the guests would have lower access levels, and the *Admin* could limit some of their devices’ functionalities, such as disabling Internet access inside that environment. The other access levels defined in UbiPri are *Blocked*, *Guest*, *Basic*, and *Advanced*.

**Figure 1:**
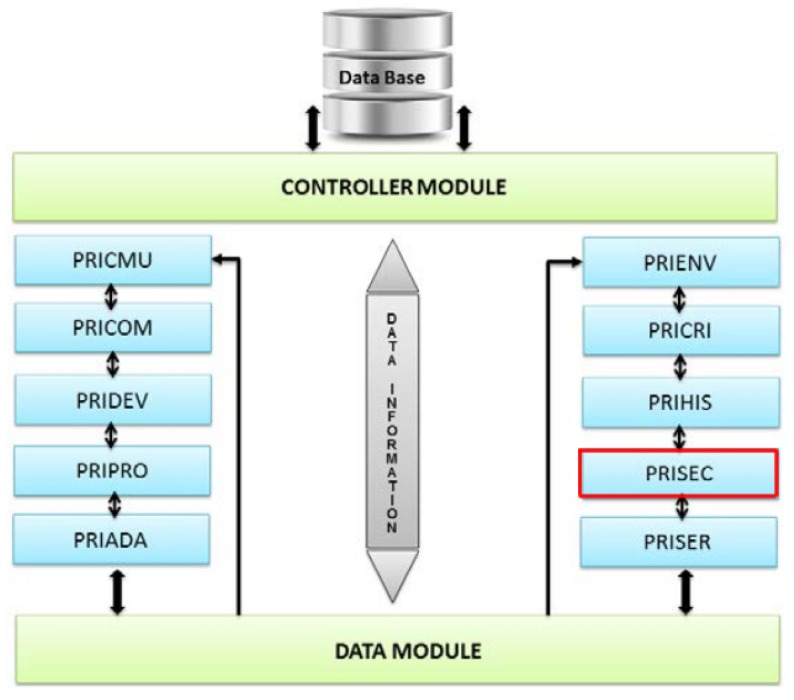
(Ubiquitous privacy) UbiPri privacy modules, Leithardt et al. [16].

The architecture of this middleware has security in mind with its PRISEC module [16]. This module controls and manages the security of its users and environments, applying the necessary cryptography and protocols to protect data. Figure 1 shows the different modules of UbiPri and how they interact with each other.

Each module is responsible for controlling and managing the privacy of different aspects of the middleware. For instance, the PRIPRO module, developed in [17,18], controls the privacy of user profiles and access levels. PRIHIS, which was developed in [19], contains the usage history of the middleware. Another module which was also developed is PRISER [20,21], managing the notifications of the users’ devices and the services of each environment.

### Motivation

The PRISEC module is still under development. On its first phase, we intend to analyze different symmetric key algorithms to determine their efficiency and apply them on the middleware based on those results. Since UbiPri will be used in an IoT context, we must choose secure cryptography algorithms while assuring fast execution times and low energy consumption. Taking into account some of the challenges and problems related to security in the IoT, and with many middleware solutions lacking security models as we have seen previously, it becomes important to have a robust cryptographic base in the middleware that we are developing. Furthermore, the tests presented here are not only relevant to our middleware but also to other systems using similar hardware and software. Thus, this paper contributes with performance evaluations of different symmetric key algorithms in IoT devices.

The chosen symmetric key block ciphers to be tested were AES, RC6, Twofish, SPECK, and LEA in GCM (Galois/counter mode) mode with all supported key sizes (128, 192 and 256 bits). For SPECK, the 128 bit block size version was chosen since the other block ciphers also use 128 bit blocks. Additionally, the authenticated encryption scheme ChaCha20-Poly1305 was included in the tests. None of these algorithms have efficient attacks published that can potentially break them, being thus considered secure. AES, RC6, and Twofish were finalists of the Advanced Encryption Standard competition, with the former algorithm winning it. SPECK and LEA are lightweight block ciphers meant to be used in resource constrained environments, being suitable for IoT devices. ChaCha20-Poly1305 is a fast stream cipher which was added to the TLS (transport layer security) 1.3 protocol, becoming thus a standard in symmetric key cryptography. Encryption time, decryption time, throughput, and power consumption will be the units to be measured.

AES is the most widely used symmetric key block cipher in computer security due to its standardization by the NIST (National Institute of Standards and Technology) and all the cryptanalysis published on this algorithm, having resisted many types of attacks. Over the years, many optimizations to its original implementation have been published, with several CPUs also supporting hardware acceleration for its operations, as is the case of the specialized AES-NI instructions. Not only does this make the algorithm more resistant to side-channel attacks, it also improves its efficiency significantly. The block size of this cipher is 128 bits, with supporting key sizes of 128, 192, and 256 bits. The number of rounds is dependent on key size, with 10 rounds for a 128 bit key, 12 rounds when using a 192 bit key, and 14 rounds for a 256 bit key. It is based on a substitution–permutation Network structure, with its main operations being *SubBytes*, *ShiftRows*, *MixColumns*, and *AddRoundKey*. The current best attack on full round AES is a biclique attack, but it is only slightly better than brute force, with the algorithm remaining secure [22].

RC6 is a symmetric key block cipher which was one of the finalists of the AES competition, being an improvement of the RC5 algorithm. Similarly to AES, it uses a 128 bit block size with key sizes of 128, 192, and 256 bits. It is based on a Feistel network, using many rotations, XOR operations and additions as its main operations. It also includes integer multiplications to increase diffusion, with the standard number of rounds being 20 [23].

Twofish was another finalist of the AES competition, being the successor of BLOWFISH. Like RC6, it is based on a Feistel network, using a 128 bit block size and supporting key sizes of 128, 192, and 256 bits. The number of rounds is 16. The best attack on full round Twofish was found with truncated differential cryptanalysis, requiring 2^51^ chosen plaintexts [24].

SPECK is one of the lightweight block ciphers developed by the NSA, along with SIMON. While SPECK is aimed at software implementations, the SIMON algorithm is intended to be used in hardware implementations. SPECK is an Add–Rotate–XOR (ARX) cipher, supporting many block and key sizes. The number of rounds is also dependent on both block and key size. The best attacks on SPECK used differential cryptanalysis, breaking around 70% of the rounds of the different SPECK variants [25].

LEA is another lightweight block cipher using an ARX design. Similarly to the AES competition algorithms, it uses a 128 bit block size and key sizes of 128, 192, and 256 bits with 24, 28, and 32 rounds, respectively. It was designed for high-speed software implementations. The work developed in [25] also applied the attack to LEA, breaking 14 rounds for 128 and 192 bit keys and breaking 15 rounds out of 32 for a 256 bit key size. Additionally, in [26], a side-channel power analysis attack allowed the retrieval of a 128 bit key in a hardware implementation of LEA. Countermeasures should be considered to avoid side channel attacks on hardware implementations of this cipher.

ChaCha20 is a high-speed stream cipher based on the Salsa20 cipher developed by Daniel J. Bernstein. These ciphers are also based on ARX operations, having 20 rounds and supporting key sizes of 256 bits. There are variants of these ciphers which use fewer rounds and a key size of 128 bits. ChaCha20 is often used with the MAC (message authentication code) Poly1305 to authenticate the encrypted messages, also developed by Bernstein. Additionally, this stream cipher was designed with side-channel cache-timing attack resistance in mind [27].

The rest of the paper is structured as follows. Section 2 discusses related works and the new performance evaluations this study brings in comparison with the research literature. Section 3 describes the test environment and the developed application to run the tests. Section 4 presents the results of the tests performed. In Section 5, we discuss the results obtained from the tests. Section 6 shows the conclusions drawn from this study. Finally, Section 7 presents the work we intend to develop in the future.

## 2. Related Works

The research literature often compared cryptography algorithms which are deemed no longer safe to use, such as DES (Data Encryption Standard), 3-DES, and BLOWFISH. These ciphers have block sizes of 64 bits which make them susceptible to collision attacks [28]. Further, some of these studies were often performed in non-IoT devices. An algorithm which is almost always present in cryptography benchmarks is AES, the standard of symmetric key cryptography, but authenticated encryption modes such as GCM are often not used. In [29], the AES, DES, and RSA (Rivest-Shamir-Adleman) algorithms were used to encrypt and decrypt medical images in tablets and smartphones, measuring their power consumption. As expected, AES obtained the best results for encryption/decryption speeds and power usage. However, the encryption mode used and key sizes were not specified.

A study made in [30] compared the execution times of the AES, DES, 3-DES, E-DES, BLOWFISH, and RSA algorithms in four messages of varying lengths. Once again, AES got the best results overall, but it would have been more interesting to compare it with more modern algorithms. The key sizes used in this study are also not clear, nor is the block cipher mode of operation specified. A similar scenario can be seen in [31], where the AES algorithm obtained a better performance than DES, RSA, and BLOWFISH. In [32], BLOWFISH got slightly better results than AES, but the latter was recommended for increased security.

In [33], the power consumption of the RC4, AES, DES, and RSA algorithms was measured on a WSN (wireless sensor network). The CBC (cipher block chaining) mode of operation was used for AES and DES. Keys of 128, 192, and 256 bits were used for AES, while for RSA, the key sizes used were 128, 256, 512, and 1024 bits. RC4 had, in general, the best power consumption, but this algorithm is no longer deemed safe due to the numerous attacks performed on it over the years [34,35,36,37]. RFC 7465 [38] also prohibited the use of RC4 in TLS. The CBC mode used for DES and AES should also be avoided since the message is not authenticated, allowing an attacker to tamper with the encrypted message.

The power consumption of the AES finalists RC6, Twofish, Serpent, and Mars was measured on an Android smartphone device in [39]. File sizes of 1, 2, 3, 4, and 5 megabytes were used for encryption and decryption of data. The Twofish and RC6 algorithms consumed the least power, followed by Mars and Serpent, respectively. Once again, key sizes and block cipher modes of operation were not specified. It is also unclear why the Rijndael algorithm, which would become the AES, was left out of the study. It would have also been interesting to have included the execution times of each algorithm to compare it with the power consumption.

The RC6, AES, 3-DES, and RSA algorithms were compared in [40], analyzing their execution time and memory used to store code, data, and constants. The RC6 algorithm obtained the best results. However, the ECB (electronic codebook) mode of operation was used, which is considered unsafe since the cipher text can leak information about the plain text due to the lack of pseudo randomness. A similar study was made in [41], comparing the RC6 and AES algorithms in ECB mode, with key sizes of 128, 192, and 256 bits. Packets of 128, 256, 512, and 1024 kB were tested. A *BeagleBone Black* device was used, which is very popular in the IoT. The RC6 algorithm got up to 10 times faster execution times in this study, but the AES hardware acceleration was disabled on the CPU of this device. It would have been interesting to show the execution times with hardware acceleration enabled and see how the RC6 execution times would compare to that.

In [42], many symmetric and public key algorithms and hash functions were tested on a *Raspberry Pi 3 Model B* and on a *Raspberry Pi Zero W*, boards commonly used in the IoT. The symmetric key algorithms included in the tests were AES in CTR (Counter) and GCM modes, using 128 and 256 bit keys, and RC6 and Twofish in CTR mode, using a 128 bit key. The performance was evaluated analyzing the throughput in MiB/second and power consumption in μWh/MiB. It was verified that RC6-128-CTR had the best throughput and power consumption in both boards in comparison with Twofish-128-CTR and AES-128-CTR. It also got better results than AES-256-CTR and AES-GCM, but the comparison here is unfair since the key sizes and mode of operation are different. With this, the study should have tested RC6 and Twofish in GCM mode with 256 bit keys as well.

Since its adoption in version 1.3 of the TLS protocol, the ChaCha20 stream cipher has been gaining the attention of security researchers. This algorithm can also achieve very fast encryption and decryption speeds, outperforming AES in CPUs without hardware acceleration. The study made in [43] shows that the authenticated encryption scheme ChaCha20-Poly1305 is faster than AES-128 in GCM, EAX, and CCM authenticated encryption modes on the ARM Cortex-M4 CPU used to run the rests, which does not have AES hardware acceleration.

The study in [44] made a quite exhaustive performance evaluation of different C/C++ cryptography libraries, among them Crypto++, Botan, OpenSSL, LibgCrypt, Nettle, and LibTomCrypt. The tested block ciphers included AES, Twofish, Serpent, Camellia, BLOWFISH, SEED, IDEA, DES, and 3-DES. Different key sizes were used, but once more, the CBC mode of operation was chosen. Pack sizes of 1, 4, and 8 megabytes were tested. The encryption and decryption speed was measured in MB/second. AES outperformed all of the algorithms due to the AES-NI instruction set, except on the LibTomCrypt library, which does not compile to the AES-NI instructions, and the Nettle library, as the authors of the study did not enable hardware acceleration support for it.

The survey made in [45] analyzed a study where the battery consumption and encryption speed of the BLOWFISH, DES, 3-DES, RC2, RC6, and AES algorithms were measured in laptops in a wireless network. Text, image, and audio files were encrypted with these algorithms. A 256 bit key was used for AES, RC6, and BLOWFISH. For DES and RC2, 64 bit keys were used. For 3-DES, the key size was 192 bits. The modes of operation are not specified for the block ciphers. BLOWFISH had the best results for text and audio files, followed by RC6. For image files, AES had better results than RC6, but DES outperformed all of the algorithms included in the study.

The time to set up the key and IV (initialization vector) and encryption speed in MiB/second of the Twofish, Camellia, Serpent, CAST-256, BLOWFISH, TEA, SHACAL-2, and Kalyna-128 were tested in an ARMv8-a CPU in [46], an architecture often used in IoT devices. A key with size 128 bits was used for all tested block ciphers in CTR mode. SHACAL-2 had the fastest encryption speed, followed by Twofish. TEA and Camellia had the lowest time to setup key and IV.

In [47], we started the performance evaluation of several symmetric key algorithms, among them AES, RC6, and Twofish, all in GCM mode. All supported key sizes were tested (128, 192, and 256 bits). However, only encryption and decryption times were measured. The tests were made in a laptop with an Intel CPU and in an emulated ARMv7-a CPU. The emulation was ran on the same laptop. We verified that AES had the best execution times for the Intel device due to hardware acceleration, but in the emulated ARMv7-a CPU, RC6 had the best results.

Recently, lightweight block ciphers have been studied frequently by researchers. These ciphers are intended to be used in resource-constrained devices, usually having simple key schedules (reducing memory requirements), running on elementary operations such as XOR or AND, and also supporting different block sizes (such as 32, 48, 64, 96, and 128 bit) [48]. Most of these lightweight ciphers are also usually targeted for either software or hardware implementations. Software-oriented lightweight cryptography includes SPECK, LEA, and Chaskey, while SIMON, LED, Piccolo, and PRESENT are among hardware-oriented lightweight ciphers [49]. Most of these ciphers have been found secure enough to be used in real world applications, with the exception of KLEIN, KTANTAN, Noekeon, and SKIPJACK, which have attacks on every or almost every round published on them and can be risky to use [48]. Otherwise, none of these ciphers are effectively broken.

In [48], many lightweight block ciphers were analyzed, among them SIMON, SPECK, HIGHT, and KATAN. Several key and block sizes were tested. The AES algorithm was also included in the study. The test device was an MSP430 16 bit microcontroller. It was verified that in software implementations, AES stood up very well to the lightweight ciphers, achieving 647 cycles per byte during encryption. SPECK with a 64 bit block size outperformed AES, with 548 cycles per byte. For a 128 bit block size, SPECK was only faster than AES during decryption. Most of the tested lightweight ciphers were also better than AES in memory usage, specially to store code and data on the stack.

A similar study was made in [49]. In addition to an MSP430 16 bit microcontroller, the tests were also ran in an 8 bit AVR and 32 bit ARM. The tested ciphers were implemented in Assembly. For encryption and decryption of 128 bytes of data in CBC mode, Chaskey was the fastest algorithm in all devices. SPECK showed some of the best results in memory usage.

In [50], the MSP430 microcontroller was once again used to test software implementations of lightweight block ciphers along with AES. TEA, XTEA, and DIRnoekeon were faster than AES for encryption and decryption. Hardware-oriented ciphers such as LED, KATAN, and PRESENT had very poor results when implemented in software.

Both hardware and software implementations were analyzed in [51]. SIMON had the overall best results for hardware implementation of the tested ciphers, with low memory requirements and decent execution times. The fastest in hardware was SEA, but it also used more memory. SPECK had the best results for software implementations.

A survey made in [52] presents a rather complete study of block ciphers, with many different algorithms and hardware and software implementations being analyzed. Ciphers like AES, Camellia, KATAN, SIMON, SPECK, and LEA were included in the survey. Hardware implementations used 0.09, 0.13, 0.18, and 0.35 μm technologies, while the software implementations were deployed in microncontrollers of 8, 16, and 32 bits. Several metrics were analyzed, including throughput and power consumption. In the hardware implementations, Piccolo got the overall best results, with SPECK, PRESENT, and TWINE being other algorithms with efficient hardware solutions. For software implementations, SPECK and PRIDE performed the best, closely followed by Fantomas, Robin, AES, and SEA.

We can see that most studies made in cryptography benchmarks have some problems, where important details about the tests were not specified or where old and unsafe ciphers were tested. Some works measured power consumption, others execution time, but few measured both. Most of these benchmarks would also only use a single test sample, providing less accurate measures. For instance, the authors of [29] tested sets of 1000 and 10,000 images, which provides more accurate results.

Table 1 shows a summary of the comparison between the research literature with the work developed in this study. If a column item is marked, the work in that row addresses it. If it is not marked, then the work either does not specify or does not address that item. The items of the table are as follows:

1. Work—Contains a reference to the study;
2. Unsafe—If the work tested unsafe ciphers. This is considered by us to be a negative factor, as the use of older and unsafe ciphers should not be motivated nor compared with modern and secure ciphers;
3. Large Samples—Whether the study used several samples to improve the accuracy of the measures;
4. Light—Informs if the study tested lightweight ciphers;
5. Key Sizes—If the work specified all of the key sizes tested and if the same key sizes were used for all algorithms, when applicable. For instance, AES and DES cannot have the same key sizes (128/192/256 bits vs. 56 bits), so in these cases, the item is marked if the key size is specified;
6. Auth Modes—Informs if the study used authenticated encryption modes for all ciphers;
7. Time—If the work tested encryption/decryption times or not;
8. PC—If power or battery consumption was measured in the work or not;
9. THP—Informs whether the study specifies encryption/decryption throughput (whether in bytes/second or cycles/byte);
10. IoT—If the tests were performed in IoT devices or not.

## 3. Test Environment and Developed Application

Since the focus of this study was evaluating the performance of symmetric key algorithms in IoT devices, the tests were performed on two smartphones with ARM CPUs, which are widely used in the IoT. These devices are also constrained energy-wise, since they depend on a limited battery. Furthermore, an Android application for the UbiPri middleware is being developed, with having the ciphers benchmarked in this platform becoming relevant.

1. Samsung Galaxy Core Prime
  - Operating System: Android 5.0.2 Lollipop
  - CPU: ARMv7-a Cortex-A7, 4 cores, 1.2 GHz
  - RAM: 1 GB
2. Xiaomi Redmi Note 3
  - Operating System: Android 6.0.1 Marshmallow
  - CPU: ARMv8-a Cortex-A53, 4 cores, 1.4 GHz + ARMv8-a Cortex-A72, 2 cores, 1.8 GHz
  - RAM: 3 GB

The Xiaomi device, having an ARMv8-a architecture, has support for AES hardware acceleration. The Samsung device does not have hardware acceleration. This way, the AES algorithm was tested on the Xiaomi device with hardware acceleration turned on and off.

**Figure 2:**
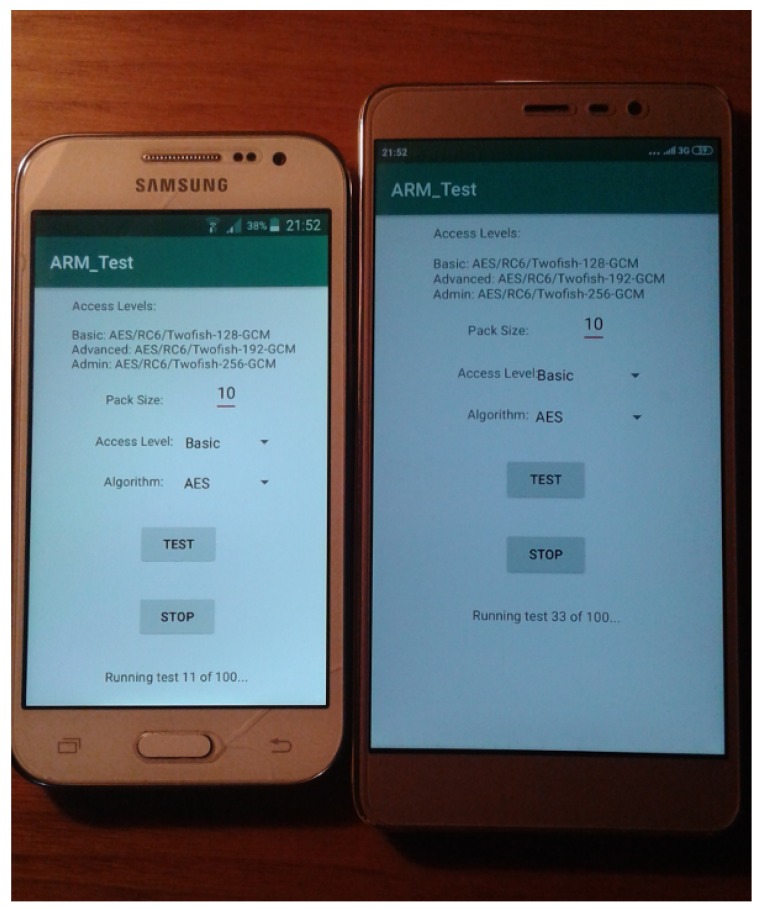
Devices used running the developed application.

An Android application was developed to run the tests. We can choose the packet size to be encrypted and decrypted, the algorithm to be used, and the access level of the user, which will determine the size of the key. The *Basic* access level uses a 128 bit key, the *Advanced* level uses a 192 bit key, and the *Admin* packets are encrypted with a 256 bit key. The block ciphers which can be chosen are AES, RC6, Twofish, SPECK128, and LEA, all in GCM mode. Additionally, the authenticated stream cipher ChaCha20-Poly1305 can be picked. Only 256 bit keys are supported for this cipher; therefore, the access level will not impact the size of the key for this algorithm. Packet sizes of 1, 5, and 10 MiB were tested for all algorithms and available key sizes. Figure 2 shows the devices used to run the tests executing the developed application for this study.

The interface was implemented in Java, while the functions which encrypt and decrypt the packets were implemented in C++ using the Android Native Development Kit. The Crypto++ 8.2 library was used since it has the implementations of all the cryptography algorithms we intended to test. It was cross-compiled to the ARMv7-a and ARMv8-a architectures with the *arm-linux-androideabi-g++* and *aarch64-linux-android-clang++* compilers, respectively. The -O3 -marm -mfpu=neon-vfpv4 compiler flags were used for the ARMv7-a compilation. When compiling for ARMv8-a, we used the -O3 -march=armv8-a+crc+simd+crypto compiler flags. To compile with the AES special instructions in the ARMv8-a device, the Crypto++ -DCRYPTOPP_ARM_AES_AVAILABLE=1 flag was also included. To compile without these instructions, and thus turning off AES acceleration, the flag -DCRYPTOPP_ARM_AES_AVAILABLE=0 was specified instead.

**Figure A1:**
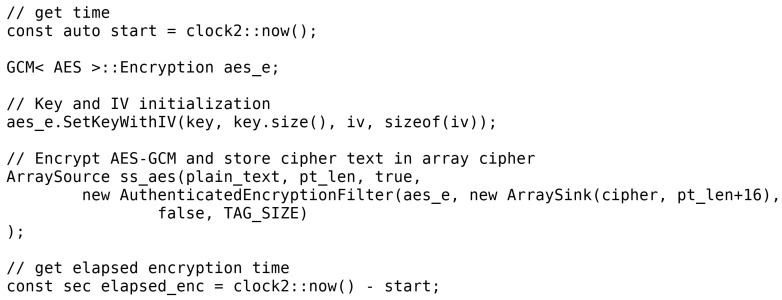
AES-Galois/counter mode (GCM) encryption with time measurement.

**Figure A2:**
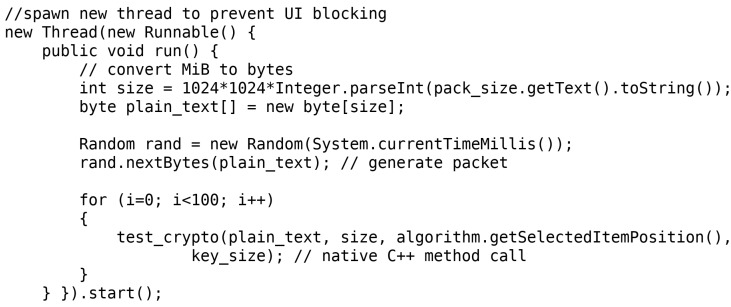
Java Backend.

A packet with the user specified size is filled with random bytes in the Java backend. The C++ method is then called passing that packet (a byte array), algorithm chosen, and access level as arguments. The packet is encrypted and decrypted 100 times, and the encryption and decryption times are measured on each run. This is done not only to warm up the CPU cache but also to get more reliable measures. A new key and IV are generated each time the packet is encrypted, but the key and IV generation time is not measured. The IV is always 12 bytes long. For ChaCha20-Poly1305, additional authenticated data (AAD) are needed. This AAD is 16 bytes long. The encryption and decryption times are measured with the <chrono> C++ library. The results are exported to a CSV file, with an average of the encryption and decryption times being obtained from them. In Appendix A, in Figure A1 and Figure A2, example codes of AES-GCM encryption time measurement and of the Java backend can be found.

**Figure 3:**
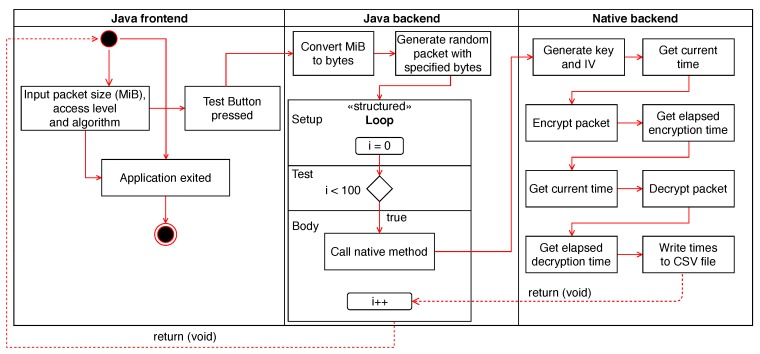
Application flow diagram.

To measure battery consumption, we used the *batterystats* dumpfile which Android provides. To get this file, the command adb shell dumpsys batterystats was run on a laptop connected to the devices. This file shows battery consumption in mAh per application. The average throughput, in MiB/s, can be obtained by dividing the encrypted/decrypted mebibytes by the encryption/decryption time. Figure 3 shows a diagram of the work flow of the developed application.

## 4. Results

### 4.1. ARMv7-a Results

**Figure 4:**
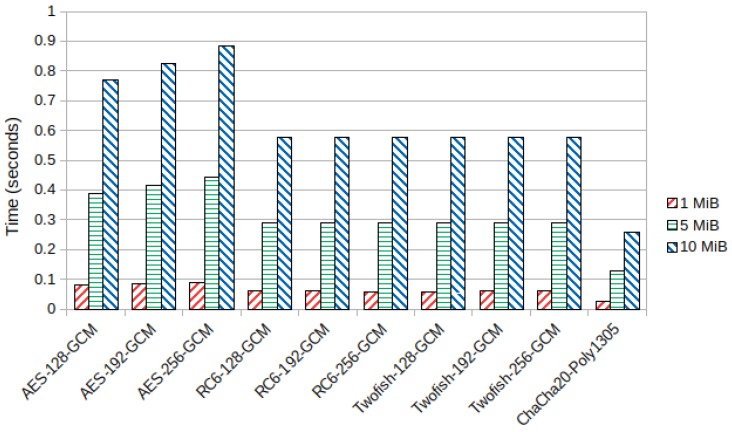
Average encryption time (seconds) in the ARMv7-a Samsung device.

For encryption and decryption times in the ARMv7-a CPU, we verified that RC6 and Twofish performed faster than AES, with the 256 bit key variants being 42% faster for a packet size of 10 MiB. Furthermore, bigger key sizes in RC6 and Twofish did not affect execution times significantly, while in AES, key size had a noticeable effect on performance. Figure 4 shows the average encryption time in seconds for AES, RC6, Twofish, and ChaCha20-Poly1305 in the Samsung device.

**Figure 5:**
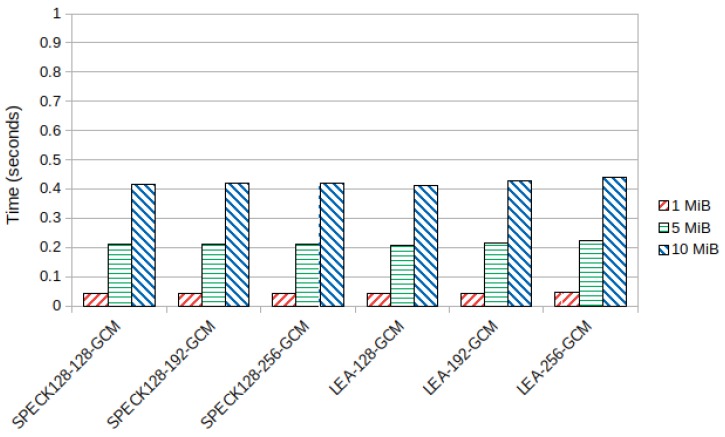
Average lightweight block cipher encryption time (seconds) in the ARMv7-a Samsung device.

**Figure A3:**
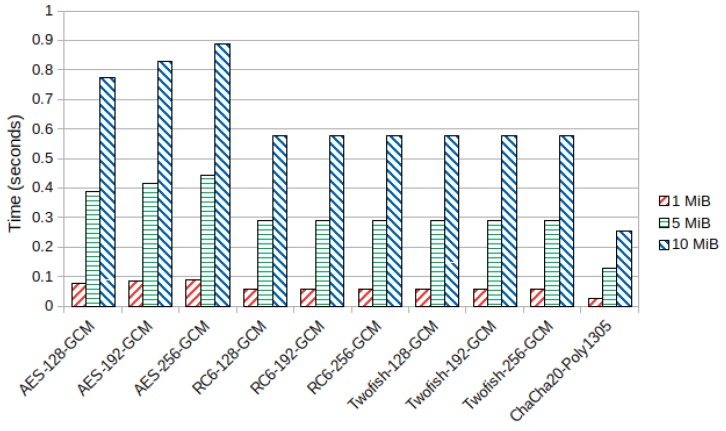
Average decryption time (seconds) in the ARMv7-a Samsung device.

**Figure A4:**
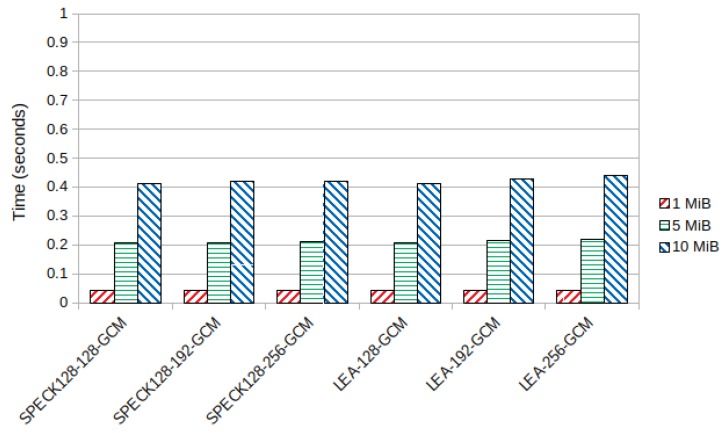
Average lightweight block cipher decryption time (seconds) in the ARMv7-a Samsung device.

The lightweight block ciphers SPECK128 and LEA performed better than the other block ciphers. SPECK128 had slightly better encryption times than LEA for key sizes of 192 and 256 bits. However, ChaCha20-Poly1305 got the overall best results, being even faster than SPECK128-128-GCM and LEA-128-GCM despite using a 256 bit key. Figure 5 shows the average encryption time in seconds for these ciphers in the Samsung device. The average decryption time, which was similar as expected from symmetric key cryptography, can be seen in Appendix B.1, Figure A3 and Figure A4.

Table 2 shows the average encryption throughput in MiB/s for each algorithm for the tested packet sizes. We can see more clearly here that Twofish had a slightly better encryption speed than RC6. However, the ChaCha20-Poly1305 authenticated stream cipher has a significant decrease in execution times, which makes it a very appealing cipher for devices with limited resources. For the average decryption throughput, see Appendix B.1, Table A1.

As mentioned in Section 3, the Android *batterystats* file was used to check the battery drain of each application. The battery consumption is presented in mAh. The Samsung device’s battery has a total capacity of 2000 mAh. Table 3 shows the battery drain for each algorithm for the given access levels. Note that this is the battery drain after running each access level test for all packet sizes. As an example, AES *Basic* shows the battery drain after running the tests for packet sizes of 1, 5, and 10 MiB. The command adb shell dumpsys batterystats --reset was executed after running such tests to reset the battery drain readings for each access level and algorithm. ChaCha20-Poly1305 only supports key sizes of 256 bits, being thus under *Admin*.

As we can see from the results, AES had the biggest battery drain. While Twofish was slightly faster than RC6, it also consumed more battery. LEA started draining more battery at access level *Advanced* (192 bit key) while being slower than SPECK128. ChaCha20-Poly1305 also had an impressive result. Not only is it faster than all other algorithms, it also consumed much less battery than the tested block ciphers.

### 4.2. ARMv8-a Results

**Figure 6:**
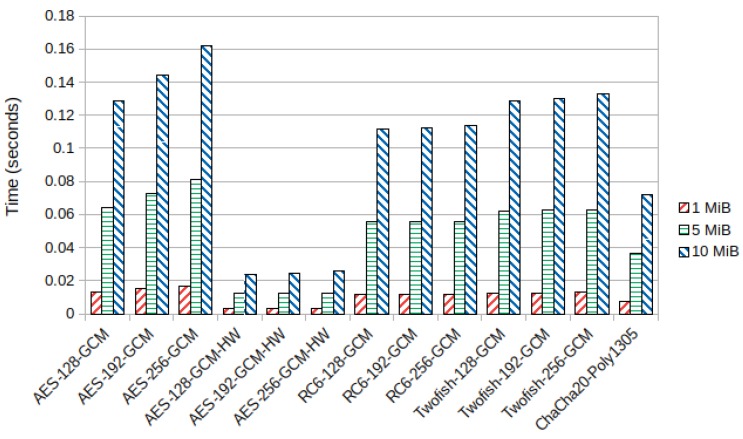
Average encryption time (seconds) in the ARMv8-a Xiaomi device.

**Figure A5:**
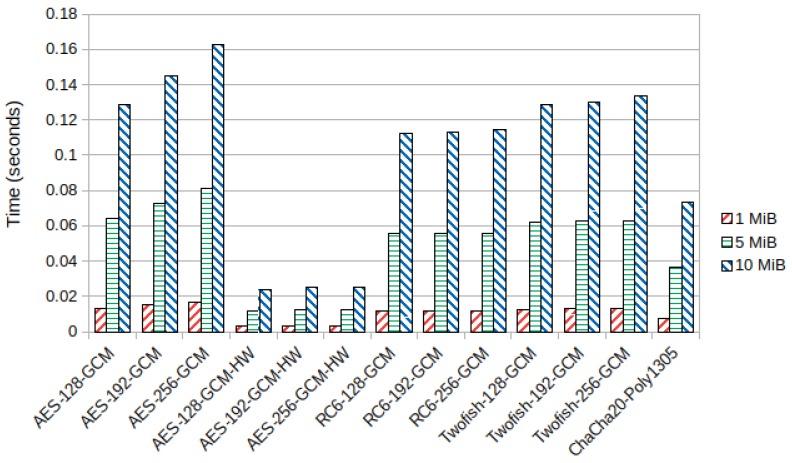
Average decryption time (seconds) in the ARMv8-a Xiaomi device.

In the ARMv8-a CPU, we got slightly different results, with RC6 being faster than Twofish. Provided hardware acceleration is off, both these block ciphers have faster encryption and decryption speeds than AES. However, with hardware acceleration turned on, AES outperformed all of the other algorithms. Figure 6 shows the average encryption time for AES, RC6, Twofish, and ChaCha20-Poly1305 in the Xiaomi device. The average decryption time, which was, once again, close to the average encryption time, can be found in Appendix B.2, Figure A5.

**Figure 7:**
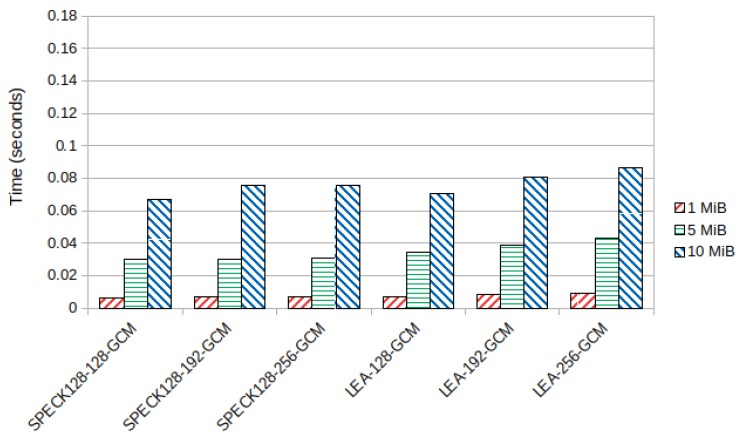
Average lightweight block cipher encryption time (seconds) in the ARMv8-a Xiaomi device.

**Figure A6:**
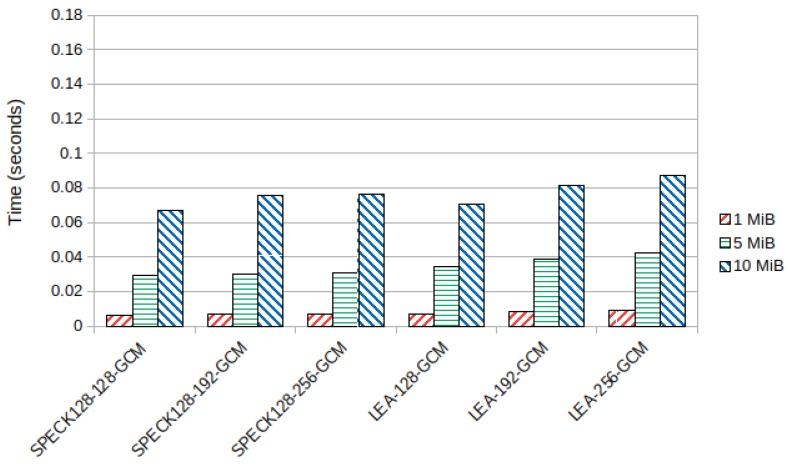
Average lightweight block cipher decryption time (seconds) in the ARMv8-a Xiaomi device.

For the lightweight block ciphers in this device, SPECK128 was always faster than LEA. Once more, both were considerably faster than the other tested block ciphers, except AES with hardware acceleration. In this device, LEA-128-GCM managed to be faster than ChaCha20-Poly1305. SPECK128 was also faster than the stream cipher, except for block sizes of 10 MiB starting at a key size of 192 bits. Figure 7 presents the average encryption time in seconds for SPECK128 and LEA in the Xiaomi device. The average decryption time can be seen in Appendix B.2, Figure A6.

Table 4 shows the average encryption throughput in MiB/s for each algorithm and packet size in the Xiaomi device. RC6 was considerably faster than Twofish. AES key size also impacted the encryption and decryption speeds much more than in the RC6 and Twofish algorithms. For the average decryption throughput in the Xiaomi device, see Appendix B.2, Table A2.

When analyzing power consumption, we verified that RC6 drained less battery than Twofish in the Xiaomi device while also having faster encryption and decryption speeds. In the Samsung device, Twofish drained more battery than RC6, but it was also faster. Without hardware acceleration, AES drained the most battery, but when using the optimized instructions, it was the most battery-efficient algorithm. SPECK128 drained less battery than ChaCha20-Poly1305 for all supported key sizes, which did not happen in the Samsung device. Table 5 shows the battery drain for all tested algorithms. The Xiaomi device’s battery has a total capacity of 4000 mAh.

## 5. Discussion

From the results described in the previous section, we can see that we have good cryptographic solutions for resource constrained devices. The results show that the CPU architecture of these devices has a considerable effect in the performance of the algorithms. For the ARMv7-a architecture, the tested lightweight block ciphers consume few resources while keeping good execution times. However, if one prefers to use one of the AES finalists instead of lightweight cryptography, either RC6 and Twofish can be good alternatives. In the emulated ARMv7-a device in [47], RC6 had faster encryption and decryption times than Twofish, which did not happen in the physical device tested here. Twofish was faster than RC6 but drained the battery slightly more.

The authenticated stream cipher ChaCha20-Poly1305 performed even better than the block ciphers, consuming less battery while being faster. It is also supported by the most recent version of the TLS protocol, along with AES, making it a robust solution security-wise.

In the ARMv8-a device, the trend verified in the Samsung device was not very similar. RC6 was up to 15% faster than Twofish, and the lightweight block ciphers managed to perform better than ChaCha20-Poly1305 in some scenarios. LEA was faster than the stream cipher for key sizes of 128 bits, while SPECK128 was faster for packet sizes smaller than 10 MiB, in addition to consuming less battery for all tested key sizes. AES-128-GCM without hardware acceleration also managed similar speeds to Twofish-128-GCM, and it drained the battery 0.05 mAh less. However, for bigger key sizes, Twofish outperformed AES without hardware acceleration.

Hardware-accelerated AES was more efficient than every other algorithm, achieving a very good encryption throughput of 426.964 MiB/s with a 128 bit key and a packet size of 10 MiB. The battery drain was also minimal, being below 1 mAh for every supported key size. From [48], we know that AES has high memory requirements, so unless our device has very limited memory resources, AES seems to be one of the best solutions in terms of speed and energy efficiency, provided the CPU has support for hardware acceleration. Otherwise, a lightweight block cipher should be used. From our tests, SPECK seems to be the overall best option when compared to LEA for a software implementation, since it was faster in most scenarios and drained less battery. SPECK also supports smaller block sizes, making it more flexible than LEA, but block sizes smaller than 128 bits should be used with care and only if the device is very constrained memory-wise to better protect against collision attacks [28]. It is also worth noting that, for block sizes other than 128 bits, the standard encryption modes of operation like GCM cannot be used as they are only defined for 128 bit block sizes. With this, other ways of authenticating the encrypted data must be explored.

## 6. Conclusions

This study has presented a more complete cryptography benchmark than previous works. Several symmetric key algorithms were evaluated with all supported key sizes and using an authenticated encryption mode. Several metrics were measured for all tested ciphers, among them execution times, throughput, and battery drain.

Care was also taken to only evaluate secure ciphers without known efficient attacks that can potentially break them. It is important to use such ciphers since they have been scrutinized over the years by the cryptography community. This not only enhances the trust we can put into any given cipher, but it also gives rise to new and more optimized implementations, saving considerable computational resources, as is the case of hardware-accelerated AES. This also gives us fewer reasons to use older and obsolete ciphers, which, while they can use fewer resources (as was the case of BLOWFISH, as seen in Section 2), are also susceptible to attacks and should be avoided. This way, modern ciphers with optimized implementations will be preferred.

## 7. Future Work

With the performance of these ciphers evaluated, we intend to implement cryptography in the UbiPri middleware based on these results. The PRISEC module will detect the characteristics of the device and decide which cipher is best in terms of security, execution times, and power consumption. The environment and access level of the user will also be considered, since access level determines the key size to be used. When it comes to the environment, the fact that it can be public or private can also have an impact on the level of cryptography to be applied, with public environments making the user’s data privacy potentially more vulnerable and thus needing stronger protection. This way, we intend to ensure the security of constrained resource IoT devices in an efficient and seamless way.

Additionally, these algorithms should also be tested in 8 bit and 16 bit microcontrollers, as the results can vary greatly from ARM CPUs as seen in [49]. The UbiPri middleware should be prepared to handle cryptography in these types of devices as they are increasingly popular in the IoT.

The CAESAR competition also introduced new authenticated cryptography solutions for many scenarios. The final portfolio announced recently in February 2019 defines three use cases. The first use case is cryptography for resource constrained environments, the second one is cryptography for high-performance applications, and the final use case is for defense in depth, with slower but stronger cryptography.

In use case 1, the finalist algorithms were Ascon and ACORN. Ascon can be implemented efficiently in hardware, being resistant to side channel attacks, and also has some degree of resistance to nonce misuse [53]. ACORN is the second choice for use case 1. Like Ascon, its focus is to be implemented efficiently in hardware, but it is also flexible enough to be implemented in software, having a small code size [54]. We hereby intend to evaluate the performance of these two authenticated encryption algorithms in several constrained resource devices, testing both hardware and software implementations with the goal of supporting them in the UbiPri middleware.

Finally, it is important to continue the research on the PRIPRO module. Since it manages the access levels of the users, it becomes an important auxiliary of the PRISEC module, as access level has a big impact on the cryptography applied to each user and environment. With the work developed in [17,18], we want to find new methods of automatically managing and assigning access levels to each user, taking into account several variables and environment characteristics. The final goal is to ensure maximum privacy and security for each user wherever the user is located and in all devices whilst consuming a minimum amount of computational resources.

## Figures and Tables

**Table 1:** Comparison of related work with the study made in this paper.

Work	Unsafe	Large Samples	Light	Key Sizes	Auth Modes	Time	PC	THP	IoT
[29]	X	X				X	X		X
[30]	X					X			
[31]	X	X		X		X			
[32]	X					X		X	
[33]	X						X		
[39]							X		X
[40]	X	X				X			X
[41]				X		X			X
[42]	X						X	X	X
[43]				X	X				X
[44]	X			X				X	
[45]	X			X			X	X	
[46]	X			X				X	X
[47]				X	X	X			
[48]			X	X				X	X
[49]			X	X				X	X
[50]			X	X				X	X
[51]			X	X			X	X	X
[52]		X	X	X			X	X	X
This work		X	X	X	X	X	X	X	X

**Table 2:** Average encryption throughput (MiB/s) in the ARMv7-a Samsung device.

Algorithm/Pack Size	1 MiB	5 MiB	10 MiB
AES-128-GCM	12.538	12.935	12.989
AES-192-GCM	11.872	12.069	12.125
AES-256-GCM	11.073	11.286	11.313
RC6-128-GCM	16.950	17.304	17.328
RC6-192-GCM	16.755	17.251	17.306
RC6-256-GCM	16.738	17.237	17.290
Twofish-128-GCM	17.094	17.324	17.372
Twofish-192-GCM	16.802	17.284	17.336
Twofish-256-GCM	16.593	17.258	17.309
SPECK128-128-GCM	23.539	23.888	24.145
SPECK128-192-GCM	23.280	23.777	23.848
SPECK128-256-GCM	22.776	23.518	23.847
LEA-128-GCM	23.801	24.242	24.214
LEA-192-GCM	22.674	23.268	23.363
LEA-256-GCM	22.125	22.523	22.677
ChaCha20-Poly1305	36.805	38.777	38.951

**Table 3:** Battery drain (mAh) in the ARMv7-a Samsung device.

Algorithm/Access Level	Basic	Advanced	Admin
AES	10.40	12.30	13.40
RC6	7.17	8.18	8.68
Twofish	7.75	8.74	8.77
SPECK128	5.59	6.16	6.32
LEA	5.18	6.49	6.65
ChaCha20-Poly1305	–	–	3.90

**Table 4:** Average encryption throughput (MiB/s) in the ARMv8-a Xiaomi device.

Algorithm/Pack Size	1 MiB	5 MiB	10 MiB
AES-128-GCM	77.539	78.058	77.586
AES-192-GCM	65.843	68.793	69.190
AES-256-GCM	59.882	61.793	61.670
AES-128-GCM-HW	325.789	414.087	426.964
AES-192-GCM-HW	300.811	411.769	409.741
AES-256-GCM-HW	299.368	399.009	391.087
RC6-128-GCM	87.089	89.975	89.785
RC6-192-GCM	86.925	89.861	88.907
RC6-256-GCM	86.615	89.799	87.939
Twofish-128-GCM	78.893	80.747	77.799
Twofish-192-GCM	78.625	79.945	77.031
Twofish-256-GCM	77.632	79.691	75.361
SPECK128-128-GCM	160.958	168.215	150.211
SPECK128-192-GCM	151.627	166.054	132.117
SPECK128-256-GCM	149.843	162.750	131.935
LEA-128-GCM	140.370	144.275	142.345
LEA-192-GCM	124.138	128.719	124.099
LEA-256-GCM	113.247	116.402	115.250
ChaCha20-Poly1305	134.081	137.656	138.336

**Table 5:** Battery drain (mAh) in the ARMv8-a Xiaomi device.

Algorithm/Access Level	Basic	Advanced	Admin
AES	2.52	2.82	3.14
AES HW	0.877	0.887	0.919
RC6	2.33	2.36	2.38
Twofish	2.57	2.63	2.73
SPECK128	1.54	1.68	1.69
LEA	1.60	1.81	1.97
ChaCha20-Poly1305	–	–	1.73

## Appendix B. Decryption Results

### Appendix B.1. ARMv7-a Decryption Results

In this appendix, the figures for the average decryption time and the table for the average decryption throughput in the Samsung device can be found. Since they were similar to the encryption results, and to prevent cluttering the main text with too many figures and tables, they were placed here.

**Table A1:** Average decryption throughput (MiB/s) in the ARMv7-a Samsung device.

Algorithm/Pack Size	1 MiB	5 MiB	10 MiB
AES-128-GCM	12.839	12.928	12.943
AES-192-GCM	12.019	12.059	12.062
AES-256-GCM	11.237	11.254	11.269
RC6-128-GCM	17.295	17.340	17.340
RC6-192-GCM	17.172	17.287	17.326
RC6-256-GCM	17.162	17.277	17.324
Twofish-128-GCM	17.304	17.364	17.385
Twofish-192-GCM	17.233	17.351	17.368
Twofish-256-GCM	17.130	17.303	17.357
SPECK128-128-GCM	23.706	24.010	24.190
SPECK128-192-GCM	23.693	23.936	23.904
SPECK128-256-GCM	23.477	23.726	23.829
LEA-128-GCM	23.953	24.251	24.244
LEA-192-GCM	23.208	23.379	23.345
LEA-256-GCM	22.579	22.679	22.734
ChaCha20-Poly1305	38.407	39.197	39.147

### Appendix B.2. ARMv8-a Decryption Results

Similarly to Appendix B.1, the figures and table of the decryption tests in the ARMv8-a Xiaomi device can be found here. For this architecture, the decryption results were also similar to the encryption process. They are nonetheless provided here.

**Table A2:** Average decryption throughput (MiB/s) in the ARMv8-a Xiaomi device.

Algorithm/Pack Size	1 MiB	5 MiB	10 MiB
AES-128-GCM	77.002	77.871	77.852
AES-192-GCM	66.671	68.541	69.026
AES-256-GCM	60.668	61.555	61.517
AES-128-GCM-HW	312.240	416.162	416.910
AES-192-GCM-HW	293.926	412.598	399.584
AES-256-GCM-HW	289.693	401.251	395.472
RC6-128-GCM	86.884	89.742	89.188
RC6-192-GCM	86.841	89.440	88.428
RC6-256-GCM	86.345	89.296	87.231
Twofish-128-GCM	78.870	80.252	77.642
Twofish-192-GCM	78.337	79.892	76.677
Twofish-256-GCM	77.980	79.794	75.040
SPECK128-128-GCM	158.101	169.930	149.765
SPECK128-192-GCM	148.910	167.075	132.916
SPECK128-256-GCM	148.604	163.716	131.265
LEA-128-GCM	137.897	145.423	142.530
LEA-192-GCM	122.688	129.230	122.834
LEA-256-GCM	112.028	117.177	114.701
ChaCha20-Poly1305	133.612	136.527	136.207
